# The Effectiveness of Anatomy Laboratory Videos on Osteopathic Medical Students’ Performance

**DOI:** 10.15694/mep.2018.0000217.1

**Published:** 2018-09-20

**Authors:** Sumathilatha Sakthi Velavan, Bedia Castellanos

**Affiliations:** 1 Marian University College of Osteopathic Medicine; 2 Touro College of Osteopathic Medicine

**Keywords:** Laboratory videos, Dissection, Anatomy, Medical Education, Technology, Students’ Perception

## Abstract

This article was migrated. The article was marked as recommended.

**Introduction:** The usage of audio-visual aids in medical education has always been rewarding. This study aimed at evaluating the efficacy of supplementing traditional dissection based laboratories with the video demonstration of specimens.

**Methods:** The study was conducted among first-year osteopathic medical students of two consecutive years. The laboratory demonstration videos were recorded and provided to the experimental group and the previous class served as the historical controls. Two Likert scale based questionnaires were completed by the experimental group before and after their final examination. The students’ performance in the Anatomy practical examinations were compared between the two groups.

**Results:** The students’ response showed that the videos added value to their knowledge. The videos helped them in understanding the structures in the dissection lab with ease they felt more confident about the examinations. The experimental group scored significantly higher grades in the practical examinations than the control group. The results confirm that the video demonstration has a positive impact on the traditional dissection method.

**Conclusion:** The effect on the student’s perception is impressive and the positive outcome in the examination grades adds on to the significance of this teaching methodology. Integration of multimedia with dissection is suggested as a helpful model to improve Anatomy learning process.

## Introduction

Anatomy is always viewed as one of the most remarkable components of medical education. Traditionally, cadaveric dissection has been the mainstream of delivering an anatomy curriculum in medical schools ( Dissabandara *et al*., 2015). Critics cite high costs, time intensity, the requirement for highly skilled teachers and the emotionally challenging nature of cadaveric dissection as potential disadvantages of cadaveric dissection ( Finkelstein and Mathers, 1990). The relevance of clinical medicine to basic sciences underwent a major re-evaluation, resulting in reduced course hours for the basic sciences ( Drake 2002). A result of medical school curricular reform has been a drastic reduction in time, form, and content of anatomic instruction and some universities no longer require dissection. The removal or attenuation of cadaver dissection is bound to impair the student’s ability to apply the scientific method during diagnosis ( Aziz *et al*., 2002).

The availability of biomedical informatics presented a convenient means of information management. The adoption of this technology in medical education was a principal goal of 80s reform ( Aziz *et al*., 2002). Following the development of digital video recording and file standards in the late 1980s and early 1990s, anatomy content began to show up in additional evolving formats namely video cassettes, video disks, CD ROMS. With continuous progressive improvements in network, data compression, and digital video format technologies, the web-distributed high-resolution video became a practical tool for broader use in integrated anatomy instruction by the mid-2000s ( Trelease, 2016). Levine *et al*., (1999) showed the potential of bringing the computer close to the dissected cadaver to allow the student to engage in an amplified learning experience. Ernst RD *et al*. (2003) digitalized and published on a website and provided a CD-ROM containing image set to medical students and faculty.

Computer technology has become an increasingly important educational resource, which has dramatically changed teaching and learning in the medical curriculum ( McNulty *et al*., 2009). Traditional teaching with lectures and dissection was supplemented with models, imaging, computer-assisted learning, problem-based learning through clinical cases, surface anatomy, clinical correlation lectures, peer teaching and team-based learning ( Trelease, 2016; Johnson, Charchanti and Troupis, 2012). The combination of computers with dissection is a natural evolution of technology and the creative teaching strategies specifically adapted for human gross anatomy laboratories in the 21st century (Reeves et al., 2012). Videos have been used for decades in teaching anatomy to practitioners in training ( Chao et al., 2010). Acland’s videos remain in active elective use by students, and a recent study reports that Australian clinical level medical students rated them very highly among available computer-assisted learning resources (Barry *et al*., 2016).

One of the more common current applications of video at the institutional level has been for capturing lectures ( Trelease, 2016). A study on streaming video for year 1-2 medical students demonstrated a positive association with program outcomes or, at a minimum, a neutral effect. It was also evident in their student survey that streaming video meets the needs of many students ( Bridge, Jackson and Robinson, 2009).

An increase in anatomy examination scores with the use of anatomy instruction videos has been reported among veterinary anatomy students ( Josephson and Moore, 2006) and medical students ( Collins *et al*., 2015). However, a study by Mahmud *et al*., (2011) showed no statistically significant difference with or without videos and the authors opined that dissection videos have the potential to become a critical resource and a partial substitute for the dissection hall itself. However, their role as a medium for learning is yet to be justified.

There is a growing belief that different educational approaches might be more appropriate and/or effective in educating physicians ( Drake, 1998). The practice of cadaver dissection has been criticized for being stressful and time-consuming. Therefore, despite its perceived effectiveness, cadaver dissection alone may not be the ideal tool for learning gross anatomy Mahmud *et al*., (2011). As a number studies on the usage of videos have had variable outcomes, the need and usage of this tool in Anatomy education should be further evaluated. In addition to analyzing the exam performance, the effect of the videos on students’ perception of approach to learning and examinations also requires an analysis. The purpose of the study was to further the existing knowledge of the efficacy of technology in the laboratory component of Anatomy by determining if the Anatomy laboratory demonstration videos facilitated learning, altered students’ perception, and their academic performance. The hypothesis tested in this study was that students taught using laboratory videos based instruction would achieve higher scores on anatomy practical examination than the students who were not taught using the videos.

## Methods

The Department of Anatomy in Touro College of Osteopathic Medicine (TouroCOM) follows a traditional cadaver-based teaching of Anatomy laboratory and flipped lectures. The course is called as ‘Clinical Anatomy and Embryology’, and it was taught over eighteen weeks during the fall semester. The course comprised of 65 lecture hours and 62 laboratory hours. All the lectures were prerecorded, posted on an online platform - ‘Blackboard, Inc.’ to which the students had authorized access. The interactive ‘Clicker sessions’ were held twice a week (34 sessions in total) which facilitated audience response based classroom discussions. The laboratory teaching was based on cadaveric dissection. The anatomy laboratory had 32 stations (each with an assigned cadaver) and the students were almost equally distributed to occupy each station. The dissection was supervised by the anatomy faculty who rotated among the stations periodically. The course material was tested in three exams. The first exam material composed of the back, upper limb and lower limb; second exam composed of thorax and head and neck; third exam composed of abdomen, pelvis and perineum. The written examination was composed of 80 board style objective questions (Bloom’s taxonomy level 2 and 3). The laboratory examination was also composed of objective questions and based on identifying 60 tagged structures that included 10 radiological images (Bloom’s taxonomy level 1).

The first year osteopathic medical students (N= 137) who had the anatomy course in 2015 participated in this study. The students (N =133) who had Anatomy the previous year in 2014 were used as historical controls. The de-identified data of both sets of students was obtained from the institution that included average age at admission, overall GPA and Science GPA and average MCAT score.

The Anatomy faculty prosected the cadavers and used them to create recordings. Adequate care was taken to cover the identity of the specimens. Each video included a demonstration of a specific region or organ. The list of videos used is included in appendix 1. The demonstrations were recorded using the Vaddio zoomSHOT 20 QUSB camera with 20X optical zoom, 1080p/60 resolution. The videos were recorded during the summer, 2015 and they were uploaded on the online platform - ‘Box.’ The students who took Anatomy in fall, 2015 were given access to these videos. Also, the corresponding videos were displayed in the laboratory at the start of every dissection session.

The students were requested to fill two anonymous and voluntary online surveys and they were made available to them as Google forms. The first survey was conducted mid-semester to analyze the impact on students’ perception of dissection after the video demonstration. Another survey was conducted after the final examination to analyze the students’ perception during their examination and preparatory phase. The questionnaires used are shown in appendix 2. Most of the questions had a five-point Likert scale with 5 being the highest and 1 being the lowest scores. The questionnaires also consisted of a column for providing any free comments. A team of Anatomists served as experts and they analyzed and validated the content of the questionnaires used in both surveys. The data obtained was used to calculate the mean, medians and interquartile range. The students’ comments were included as a qualitative component of the study. The second survey had a question that asked for the number of times the students viewed the videos before they took their practical examinations. A question asked if the students wanted similar videos to be included in Neuroanatomy. The surveys were used to analyze the effectiveness of the videos in facilitating the students’ learning process and also to assess the students’ perception.

The practical examination grades of the test and historic control groups were used to analyze the impact of the laboratory videos on the academic performance of students. The data sets were de-identified and the overall class performance of the two student groups was compared using two-sample t-tests. The two student groups were longitudinally followed up to track their Anatomy scores in COMLEX-USA level 1 examination. A group of 119 students from the control group and 130 students from the experimental group took their COMLEX examination in the years 2016 and 2017 respectively.

The research proposal was approved by Touro University’s Human Subjects Institutional Review Board and the board ruled that the study was exempt, due to it being an evaluation of an educational technique (protocol number HSIRB 1647E).

## Results

The student population of the two groups was comparable in their average age, MCAT scores, GPA ( table 1). The P-values indicated that the two groups are not statistically different in their academic standards. The two classes had the same lecture and laboratory hours and they were tested by the examinations of the same level of difficulty as indicated by the same number of questions of the same Bloom’s Taxonomy category.

**Table 1.** Demographics

	2014 group (Control) N= 133	2015 group (Experimental) N=137	P value
**Average age (years)**	25.9 (+/- 3.41)	25.52 (+/- 3.42)	0.4
**Overall GPA**	3.42 (+/- 0.22)	3.43 (+/- .25)	0.7277
**Science GPA**	3.31 (+/- ).26)	3.36(+/- .26)	0.1153
**MCAT total**	28.11 (+/- 4.15)	28.20(+/- 4.57)	0.8657

The first survey showed a participation rate of 64% and the summary of students’ response showed that majority of the students perceived that the videos allowed better understanding of the dissection; encouraged to perform better dissection; provided student autonomy and to be less dependent on the instructor in the laboratory; enabled more efficient utilization of the laboratory time; reduced the stress related to learning. The majority of the students preferred to have similar sessions in the future ( table 2). The second survey had a participation rate of 47% Most of the students watched the videos three times before the exam; felt confident about the practical exam; had reduced stress level during the exam ( table 3). The analysis of both the surveys showed the interquartile range to be one to two. All the responders felt that laboratory videos should become a part of teaching Neuroanatomy also.

The comment section also showed that the students were better able to locate the structures given in the examination review sheet during their personal review time. Some of the comments were ‘The videos supplemented my studying and allowed me to be better prepared for the practical,’ ‘it helped me out tremendously,’ ‘Very helpful for practical preparation,’ ‘The lab videos were a great addition to the class,’ ‘Having the videos made before lab is SO BENEFICIAL to actually knowing how to dissect,’ ‘I really like the video presentations. They are very informative. These videos could serve as quick reviews before practical exams,’ ‘They stimulated a lot of discussion in our group and clarified a lot of concepts so we did not have to walk around and look for an instructor.’

**Table 2.** Analysis of Survey 1

Questions	Mean	Median	Interquartile range
The learning objectives were met.	4.425 (+/- 1.06)	5	1
The teaching methodology was stimulating and engaging.	4.402 (+/- 1.04)	5	1
The demonstration allowed better understanding of the high yield concepts.	4.494 (+/- 1.06)	5	1
The videos measured what I had already learned of that region	4.022 (+/- 1.02)	4	1.5
The videos encouraged me to perform better dissections.	4.255 (+/- 0.94)	4	1
The videos allowed me to achieve my dissection goals.	4.172 (+/- 0.96)	4	1
The videos provided student autonomy and to be less dependent on the instructor in the lab.	3.885 (+/- 1.05)	4	2
The video reduced the conflict of ideas among the students within the lab group.	3.758 (+/- 1.01)	4	2
The videos created a learning environment that allowed for questions and discussions while lab was in session.	4 (+/- 0.94)	4	2
The video enabled more efficient utilization of the lab time.	4.287 (+/- 0.91)	4	1
The video helped me remember key information and anatomical landmarks.	4.581 (+/- 0.83)	5	1
The demonstration of the structures added value to my knowledge of the structures.	4.581 (+/- 0.81)	5	1
The teaching method made me feel more confident about the practical exam.	4.379 (+/- 0.91)	5	1
The demonstration reduced the stress associated with learning.	4.252 (+/- 0.94)	4	1
I would like to have similar sessions in the future.	4.655 (+/- 0.84)	5	0

**Table 3.** Analysis of Survey 2

Questions	Mean	Median	Interquartile range
The quality of videos was good.	4.140	4 (+/- 0.75)	1
The volume of material was appropriate for the topic.	4.234	4 (+/- 0.58)	1
Some labs were preceded by video demonstration. This helped me to be better prepared for the lab.	3.546	4 (+/- 1.18)	1.25
I felt confident about the practical exam.	4.08	4 (+/- 0.74)	1
My stress level reduced during the exam	4.34	4 (+/- 0.7)	1

**Figure 1.**
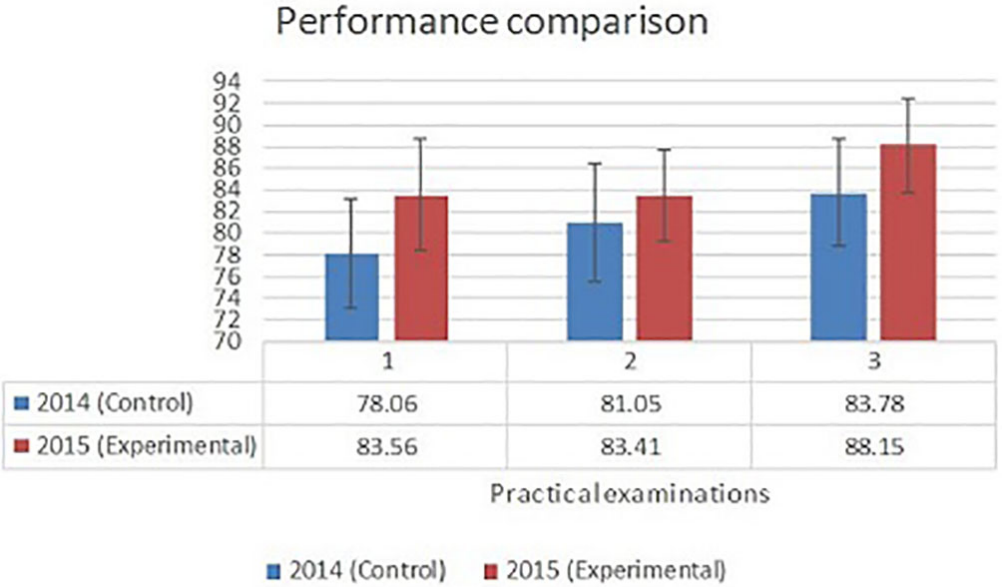
Anatomy practical examination comparison

Anatomy practical examination scores of the experimental group were consistently higher in all the three practical examinations compared to the scores of the control group. A two-sample t-test was conducted using Graph pad software to compare the performance of the two groups of students in anatomy practical examinations. The class average showed a statistically significant difference (P < 0.001) in the Anatomy practical examination scores between the two groups ( figure 1).

The two groups of students were followed through their COMLEX level 1 exam. Their class average of the Anatomy score was compared and the P value was 0.0034 and this difference was very statistically significant ( table 4).

**Table 4.** COMLEX scores comparison

	2014 group (Control) N=119	2015 group (Experimental) N= 130)	P value
COMLEX Anatomy Average score	535.29 (+/-115.38)	579.32 (+/- 119.18)	00.0034

## Discussion

Anatomy educators at TouroCOM have been focusing on modifying the teaching methodology to suit the students’ and curricular requirements. Being tasked with delivering the vast material in the face of fewer laboratory hours, they have turned to technology-based education to supplement delivery of relevant material. The improved students’ performance reflects a shift in teaching style at TouroCOM.

The video demonstration has been found to have a lot of benefits in various other studies. Another study showed that the use of video material as an introductory learning tool in gross anatomy reduced anticipatory anxiety responses in medical students (Casado, Castaño and Arráez-Aybar, 2012). The high rating of the laboratory videos by the students and their comments revealed that the structures were much easier to identify and the laboratory hours were utilized more appropriately. Usually, there is a difference of opinion among students of the same station in the identification of the structures. The survey showed that laboratory videos diminished such conflicts among students. Every faculty has a different style of teaching and the volume of information shared with the students by each faculty is also variable. In the process of repetitive teaching to different student groups, faculty might inadvertently miss few details. The addition of the laboratory videos created uniformity in the demonstration of laboratory content. Prior to the advent of these videos, TouroCOM faculty conducted additional laboratory review sessions when requested by the students and the faculty always found it difficult to engage all the students. Some students usually felt left out, since they could not attend such sessions or due to the difficulty in visualizing the structures being in a group. Such ‘on-demand’ based laboratory review sessions drastically went down in number. The students could watch the videos any number of times at their own convenience.

An overall improvement in practical examination grades from exam 1 to exam 3 was observed in both the groups. This is a usual trend observed as the students get more familiar with the curricular content and examinations. The experimental group scored higher than the control and the difference in academic performance was statistically significant in a consistent manner in all the examinations. Since the two groups of students matched in their demographic data and curricular content, the difference is most likely the outcome of the laboratory videos. A similar study by Topping *et al.,* (2014) utilized students from the prior batch as historic controls and there was no significant difference in the overall final grades, however, a significant improvement in score on the laboratory final examination. In this study, the videos may have served as a useful adjunct to the restrictions that were placed on the curriculum. The material that was covered on the videos was previously given in a teaching station scenario, where the instructor guided small groups through the same material given in the video. It is quite possible that the videos were just as good as the guided prosection sessions given in the previous year Topping *et al.,* (2014).

Research on the satisfaction and use of anatomy videos on examination performance continues to grow ( Bridge, Jackson and Robinson, 2009; Topping *et al.,* 2014). Tam *et al*., (2009) revealed that computer-aided instruction may be useful in bridging the gap created by a reduction in gross anatomy course hours. Engaging and entertaining solutions that deliver high-quality information should be sought out to motivate the medical students to master relevant subjects. It stands a reason that a positive experience will help them retain the material and eventually deliver better patient care (McNulty, Halama and Espiritu, 2004; Topping *et al.,* 2014). Studies by DiLullo *et al., (2006)* and Bee *et al*., (2012) on digital video clips to present dissection guidance to medical students found increased students satisfaction and significantly better performance. Survey respondents indicated that the videos enhanced the quality of the anatomy course as well as their individual performances.

Two other studies inferred that the students with and without access to the videos did not differ in examination performance ( Saxena *et al*., 2008; Mahmud *et al*., 2011). Nevertheless, in a regression analysis controlling for age and MCAT scores, using the anatomy videos at least once improved anatomy examination performance by 3.4% in one of the studies ( Saxena *et al*., 2008). Also, the other study showed a small, nonstatistically significant increase in anatomy term test scores. Almost half the students regarded the dissection videos as the best resource for learning anatomy ( Mahmud *et al*., 2011). A study that evaluated the impact of video demonstrations eliminating cadaveric dissection in a veterinary medical school showed that cadaver prosections were superior to video demonstrations and inferred that dissection videos should not replace cadaver prosection ( Theoret, Carmel and Bernier, 2007).

Gross anatomy through dissection and prosection cannot be undermined in a modern medical curriculum since it gives a 3D experience in real life that cannot be attained by the most advanced digital anatomy programs available. The new digital tools and the new PBL and integrative teaching methods have to be ancillary and complement the gross anatomy education and the lecture experience ( Papa and Vaccarezza, 2013). Cadavers and computers complement each other and, when combined provide the best results (Biasutto, Caussa and Criado del Rıo, 2006). Studies based on laboratory videos are not limited to the first year medical students. The video recordings have also been used in clinical teaching. A study by Abdelsattar *et al., (2015)* showed the effectiveness of creating a 4-minute video clip by experienced surgeons that was used to relay surgical facts to trainees. YouTube has publicly available teaching videos, but preliminary studies investigating its utility in learning surface anatomy have indicated a current lack of appropriate quality content ( Azer, 2012). Although plenty of other web-based resources are available, the videos specifically designed and created for the students by their own faculty based on the dissection schedule of an institution is highly valuable.

A few limitations of this study were however noted. An ideal study should have had the students of the same batch divided into two groups, exposing only one group to laboratory videos and comparison of the examination results of two groups in the same exam. However, the researchers did not want to deprive any of the students of the benefits of having the laboratory videos. Hence the entire batch was given access to the videos. The other limiting factor was that the participation of students in the survey was not 100%. One more limiting factor was that the platform used to upload the recorded videos lacked tracking facility and hence the frequency at which the students accessed the resource could not be tracked. Also, the study did not measure the individual student’s usage of videos and its relation to his/her academic performance.

## Conclusion

A general conclusion is that computer-aided instruction is equal to, and sometimes better than, conventional methods of teaching in a level of student satisfaction and knowledge gains. Moreover, computer applications have improved efficiencies by providing more controlled environments with opportunities for adaptive and collaborative learning ( McNulty *et al.,* 2009). The study was conducted as a first step at TouroCOM and the results were promising. The students and faculty felt that these videos are perfect inclusion in the existing curriculum. Moving forward, TouroCOM planned to make an entire video library of Anatomy dissection for the first year students. A short introduction to dissection technique and a demonstration of the relevant structures add a significant advantage to alleviate the stress of medical students and improve their academic performance.

## Take Home Messages

• The medical students are highly satisfied with the inclusion of laboratory videos in anatomy education
• The laboratory recordings facilitated cadaveric dissection by the students
• The students felt confident about their dissection and preparation for the anatomy examination
• The academic performance is significantly better when dissection was supplemented by laboratory videos.

## Notes On Contributors

Sumathilatha Sakthi Velavan, MBBS, DGO, MS is an assistant professor of anatomy in the Department of Biomedical Sciences at Marian University College of Osteopathic Medicine. The study was conducted when she worked at Touro College of Osteopathic Medicine. She teaches Anatomy, Histology, and Embryology to medical students. Her research interests are in anatomical variations and medical education.

Bedia Castellanos, MD, is an instructor in the Department of Anatomy at the Touro College of Osteopathic Medicine in New York, New York. She teaches anatomy and Neuroanatomy to first-year medical students and her research interest is in anatomical variations and technology-based medical education.

## Appendices

### Appendix 1

List of laboratory recordings:

Back and Upper limb

• Superficial back
• Deep back
• Pectoral region and shoulder
• Arm
• Forearm
• Hand

Lower limb

• Front and medial compartments of thigh
• Gluteal region and back of thigh
• Front and lateral compartment of leg
• Popliteal fossa and posterior compartment of leg
• Dorsum and sole

Thorax

• Pericardium and external features of heart
• Internal features of heart
• Blood supply of heart
• Pleura and lung
• Posterior mediastinum

Abdomen and Pelvis

• Anterolateral abdominal wall
• Inguinal canal, Scrotum
• Peritoneum and peritoneal cavity, Viscera in situ
• Stomach and celiac trunk
• Small intestines, SMA
• Large intestines, IMA,
• Liver, Pancreas, Spleen
• Posterior abdominal wall
• Kidneys, Ureter, Suprarenal gland
• Bladder, Rectum
• Male pelvic viscera
• Female pelvic viscera
• Perineum

Head and Neck

• Superficial structures of the neck
• Deep structures of the neck
• Prevertebral region
• Scalp, Face and parotid
• Temporal region, Infra temporal Fossa
• Cranial cavity
• Orbit
• Nose, Oral cavity, Pterygopalatine fossa

### Appendix 2

Questionnaires used:

Survey 1

• The learning objectives were met
• The teaching methodology was stimulating and engaging
• The demonstration allowed better understanding of the high yield concepts
• The videos measured what I had already learned of that region
• The videos encouraged me to perform better dissections
• The videos allowed me to achieve my dissection goals
• The videos provided student autonomy and to be less dependent on the instructor in the lab
• The video reduced the conflict of ideas among the students within the lab group
• The videos created a learning environment that allowed for questions and discussions while lab was in session
• The video enabled more efficient utilization of the lab time
• The video helped me remember key information and anatomical landmarks
• The demonstration of the structures added value to my knowledge of the structures
• The teaching method made me feel more confident about the practical exam
• The demonstration reduced the stress associated with learning
• I would like to have similar sessions in the future
• Please add any suggestions to improve:

Survey 2

• The quality of videos was good
• The volume of material was appropriate for the topic
• The labs were preceded by video demonstration. This helped me to be better prepared for the lab
• I felt confident about the practical exam
• My stress level reduced during the exam
• I would like to have lab videos in the future for Neuroanatomy
• Further comments

## References

[ref1] Abdelsattar J. M. Pandian T. Finnesgard E. J. El Khatib M. (2015) Do You See What I See? How We Use Video as an Adjunct to General Surgery Resident Education. Journal of Surgical Education. 72(6), pp.145–150. 10.1016/j.jsurg.2015.07.012 26454723

[ref2] Azer S. A. (2012) Can “YouTube” help students in learning surface anatomy? Surgical and Radiologic Anatomy. 34(5), pp.465–468. 10.1007/s00276-012-0935-x 22278703

[ref3] Aziz M. A. Mckenzie J. C. Wilson J. S. Cowie R. J. (2002) The human cadaver in the age of biomedical informatics. The Anatomical Record. 269(1), pp.20–32. 10.1002/ar.10046 11891622

[ref4] Barry D. S. Marzouk F. Chulak-Oglu K. Bennett D. (2015) Anatomy education for the YouTube generation. Anatomical Sciences Education. 9(1), pp.90–96. 10.1002/ase.1550 26061143

[ref5] Bee M. Montante J. and McAuley R. (2012) Cadaver dissection videos as an effective lab instruction tool for medical and other graduate level health professional students. Proceedings of the Experimental Biology 2012, The FASEB J. 26(1), supplement, 530.9. Available at: https://www.fasebj.org/doi/abs/10.1096/fasebj.26.1_supplement.530.9

[ref6] Bridge P. Jackson M. and Robinson L. (2009) The Effectiveness of Streaming Video on Medical Student Learning: A Case Study. Medical Education Online. 14(1). 10.3402/meo.v14i.4506 PMC2779626 20165525

[ref7] Casado M. I. Cstaño G. and Arráez-Aybar L. A. (2011) Audiovisual material as educational innovation strategy to reduce anxiety response in students of human anatomy. Advances in Health Sciences Education. 17(3), pp.431–440. 10.1007/s10459-011-9307-2 21678089

[ref8] Chao T. Wendel G. McIntire D. and Corton M. (2010) Effectiveness of an instructional DVD on third- and fourth-degree laceration repair for obstetrics and gynecology postgraduate trainees. International Journal of Gynecology & Obstetrics. 109(1), pp.16–19. 10.1016/j.ijgo.2009.10.016 20022005

[ref9] Collins A. M. Quinlan C. S. Dolan R. T. O’Neill S. P. (2015) Audiovisual preconditioning enhances the efficacy of an anatomical dissection course: A randomized study. Journal of Plastic, Reconstructive & Aesthetic Surgery. 68(7), pp.1010–1015. 10.1016/j.bjps.2015.03.010 25865740

[ref10] DiLullo C. Coughlin P. D’Angelo M. McGuinness M. (2006) Anatomy in a New Curriculum: Facilitating the Learning of Gross Anatomy using Web Access Streaming Dissection Videos. Journal of Visual Communication in Medicine. 29(3),99–108. 10.1080/01405110601080738 17162338

[ref11] Dissabandara L. O. Nirthanan S. N. Khoo T. K. and Tedman R. (2015) Role of cadaveric dissections in modern medical curricula: a study on student perceptions. Anatomy & Cell Biology. 48(3), pp.205–212. 10.5115/acb.2015.48.3.205 26417481 PMC4582164

[ref12] Drake R. (2002) Meeting the challenge: The future of the anatomical sciences in medical school curricula. The Anatomical Record. 269(2), pp.68–68. 10.1002/ar.10082 12001212

[ref13] Drake R. L. (1998) Anatomy education in a changing medical curriculum. The Anatomical Record. 253(1), pp.28–31. 10.1002/(SICI)1097-0185(199802)253:1<28::AID-AR11>3.0.CO;2-E 9556023

[ref14] Ernst R. D. Sarai P. Nishino T. Hernandez A. (2003) Transition from film to electronic media in the first-year medical school gross anatomy lab. Journal of Digital Imaging. 16(4), pp.337–340. 10.1007/s10278-003-1700-9 14749968 PMC3044075

[ref15] Finkelstein P. and Mathers L. (1990) Post-traumatic stress among medical students in the anatomy dissection laboratory. Clinical Anatomy. 3(3), pp.219–226. 10.1002/ca.980030308

[ref16] Johnson E. Charchanti A. and Troupis T. (2012) Modernization of an anatomy class: From conceptualization to implementation. A case for integrated multimodal-multidisciplinary teaching. Anatomical Sciences Education. 5(6), pp.354–366. 10.1002/ase.1296 22730175

[ref17] Josephson E. and Moore L. (2006) An Electronic Instructor for Gross Anatomy Dissection. Journal of Veterinary Medical Education. 33(3), pp.465–473. 10.3138/jvme.33.3.465 17035225

[ref18] Levine M. Stempak J. Conyers G. and Walters J. (1999) Implementing and integrating computer‐based activities into a problem‐based gross anatomy curriculum. Clinical Anatomy. 12(3), pp.191–198. 10.1002/(SICI)1098-2353(1999)12:3<191::AID-CA8>3.0.CO;2-5 10340460

[ref19] Mahmud W. Hyder O. Butt J. and Aftab A. (2011) Dissection videos do not improve anatomy examination scores. Anatomical Sciences Education. 4(1), pp.16–21. 10.1002/ase.194 21265032

[ref20] McNulty J. A. Halama J. and Espiritu B. (2003) Evaluation of computer-aided instruction in the medical gross anatomy curriculum. Clinical Anatomy. 17(1), pp.73–78. 10.1002/ca.10188 14695594

[ref21] McNulty J. Hoyt A. Gruener G. Chandrasekhar A. (2009) An analysis of lecture video utilization in undergraduate medical education: associations with performance in the courses. BMC Medical Education. 9(6). 10.1186/1472-6920-9-6 PMC2647683 19173725

[ref22] Papa V. and Vaccarezza M. (2013) Teaching Anatomy in the XXI Century: New Aspects and Pitfalls’, The Scientific World Journal, Article ID 310348. 10.1155/2013/310348 PMC3842041 24367240

[ref23] Reeves R. Aschenbrenner J. Wordinger R. Roque R. (2004) Improved dissection efficiency in the human gross anatomy laboratory by the integration of computers and modern technology. Clinical Anatomy. 17(4), pp.337–344. 10.1002/ca.10245 15108341

[ref24] Saxena V. A. Natarajan P. O’Sullivan P. S. and Jain S. (2008) Effect of the use of instructional anatomy videos on student performance. Anatomical Sciences Education. 1(4), pp.159–165. 10.1002/ase.38 19177403

[ref25] Tam M. Hart A. Williams S. Heylings D. (2009) Is learning anatomy facilitated by computer-aided learning? A review of the literature. Medical Teacher. 31(9), pp.393–396. 10.1080/01421590802650092 19811174

[ref26] Theoret C. L. Carmel É. and Bernier S. (2007) Why Dissection Videos Should Not Replace Cadaver Prosections in the Gross Veterinary Anatomy Curriculum: Results from a Comparative Study. Journal of Veterinary Medical Education. 34(2), pp.151–156. 10.3138/jvme.34.2.151 17446641

[ref27] Topping D. B. (2013) Gross anatomy videos: Student satisfaction, usage, and effect on student performance in a condensed curriculum. Anatomical Sciences Education. 7(4), pp.273–279. 10.1002/ase.1405 24106107

[ref28] Trelease R. (2016) From chalkboard, slides, and paper to e-learning: How computing technologies have transformed anatomical sciences education. Anatomical Sciences Education. 9(6), pp.583–602. 10.1002/ase.1620 27163170

