# Supplementary material for: The Effectiveness of Anatomy Laboratory Videos on Osteopathic Medical Students’ Performance
Source: MedEdPublish (2016). 2018 Sep 20;7:217. [Version 1] doi: 10.15694/mep.2018.0000217.1 (PMC10704422; doi:10.15694/mep.2018.0000217.1)
Supplement: Peer review history

# Reviewer report: Reviewer response for version 1

**DOI:** 10.21956/mep.18986.r27210

**Published:** 2019-02-22

**Authors:** Trevor Gibbs

**Affiliations:** 1 AMEE

This review has been migrated. The reviewer awarded 4 stars out of 5 An interesting paper that explores an active teaching method to a recognised basic science learning situation.The authors have outlined their project well, and very clear and have pointed out the relevant limitations to the study. I too would have liked to see more students from different schools participating in this to increase the numbers slightly, but my one issue was the lack of a more longitudinal evaluation, rather than basing the positives on an assessment procedure i.e. did it affect / improve their clinical learning? I did feel however that it was a paper worthwhile reading for those involved in anatomy teaching.

Reviewer Expertise:

NA

No decision status is available

# Reviewer report: Reviewer response for version 1

**DOI:** 10.21956/mep.18986.r27211

**Published:** 2018-10-21

**Authors:** Ken Masters

**Affiliations:** 1 Sultan Qaboos University

This review has been migrated. The reviewer awarded 4 stars out of 5 Overall, a well-conducted study, and well-written paper, showing the value of using specially-constructed videos in teaching, taking into account both student perceptions and impact of performance. A limitation that should be noted is that this study was conducted at a single institution under specific circumstances, and so generalisability cannot be assumed. Nevertheless, this is a useful platform on which to build further research, for the authors and other researchers. As a personal interest area, I would like to have seen if the researchers had checked the accesses (either through server logs, or general observing of the students), to get a sense of what systems (desktop machines, laptops, tablets or other devices) the students were using to access the videos. I don’t wish the authors to have to include this information in a revision of their paper, as it was not part of their aim, but, if you could reply to this review here, that would be useful.

Reviewer Expertise:

NA

No decision status is available

# Reviewer report: Reviewer response for version 1

**DOI:** 10.21956/mep.18986.r27212

**Published:** 2018-09-25

**Authors:** Bengang Qin

**Affiliations:** 1 The First Affiliated Hospital of Sun Yat-sen University

This review has been migrated. The reviewer awarded 4 stars out of 5 This is a very interesting paper and presents the effectiveness of anatomy laboratory videos on osteopathic medical students’ performance. The text is well arranged and the logic is clear. There are some three comments I have.1. Laboratory videos being used to teach are very population in medical school. Moreover, there are little controversy about the effectiveness of anatomy laboratory videos on osteopathic medical students performance. Therefore, there is insufficient novelty.2. The sentence of the conclusion depart “Moreover, computer applications have improved efficiencies by providing more controlled environments with opportunities for adaptive and collaborative learning (McNulty et al., 2009)” should be deleted. It is not your conclusion.3. Some of literature are older and recent three years literatures need to be added. I would like to re-review the paper in great depth once it has been edited because the subject is interesting.

Reviewer Expertise:

NA

No decision status is available
